# An Overview of Emerging Trends in Targeted Therapy of Triple‐Negative Breast Cancer

**DOI:** 10.1155/ijbc/8575520

**Published:** 2026-07-22

**Authors:** Anuj Kumar, Suresh Subramanian

**Affiliations:** ^1^ Radiopharmaceuticals Division, Bhabha Atomic Research Centre, Mumbai, India, barc.ernet.in; ^2^ Homi Bhabha National Institute, Mumbai, India, hbni.ac.in

**Keywords:** antibody–drug complex, breast cancer, targeted therapy, TNBC, triple-negative breast cancer

## Abstract

Triple‐negative breast cancer (TNBC) is a biologically diverse, highly aggressive class of breast cancer defined by the absence of estrogen, progesterone, and HER2 receptors. It accounts for approximately 10%–20% of all invasive breast cancers, and disproportionately affects women of younger ages and from minority groups. TNBC is distinguished by delayed diagnosis requiring special techniques, rapid metastatic progression, and limited treatment options compared with other breast cancers. Chemotherapy remains the mainstay but patients face higher relapse rates and overall survival is significantly reduced. TNBC represents a diverse array of subtypes, whose molecular differences impact their pathological behavior and response to therapy. Newer molecular markers in various stages of clinical and preclinical development show promise towards improving the management of TNBC patients. Membrane receptor proteins like trop2, nectin‐4, LIV‐1, gpNMB, CXCR4, DDR1, and PD‐L1 have been targeted with antibody–drug complexes. Synthetic small molecules and antisense oligonucleotides have been employed for inhibition of internal cellular components like PARP enzyme and expression of genes related to cancer progression. The molecular makeup of the tumor microenvironment is also a significant factor in addressing TNBC metastasis. In conclusion, the stratification of TNBC patients on their molecular subtype needs to be more widely adopted, and a calculated combination of current and emerging strategies is needed to effectively address TNBC in the clinic.

## 1. Triple‐Negative Breast Cancer (TNBC) as an Important Subset of Breast Cancer

### 1.1. Epidemiology and Risk Factors

Breast cancer has the second most common incidence across sexes (~2.3 million new cases as per Globocan 2022, 25% of total cancers) and is the fourth common cause of cancer mortality worldwide (~0.67 million deaths). In women, it is the most widely diagnosed cancer and the top cancer mortality cause. By 2050, breast cancer incidence is expected to increase 38% and mortality by 68%, heterogeneously impacting less developed nations [1].

Early diagnosis and treatment are key to better prognosis in breast cancer. Markers like estrogen receptor (ER), progesterone receptor (PR), or human epidermal growth factor receptor 2 (HER2) have differential expression in the majority of breast cancers and have been employed in the diagnosis and therapy of patients. However, the American Society of Clinical Oncology/College of American Pathologists warns that 15%–20% of newly diagnosed breast cancer cases have negligible expression of all these markers. These are denoted as TNBC [2].

TNBC cases present a significantly more unfavorable patient outcome within the overall breast cancer patient pool due to (a) limitations of early diagnosis techniques and (b) their tendency to form secondary metastases in the brain, liver, and lungs. Presently, TNBC has limited treatment options; in the United States, it carries a 5‐year survival rate of < 15% for patients with distal metastases [3].

Almansour′s 2022 review [4] systematically covers risk factors for TNBC: age, ethnicity, and genetic predisposition rank among the nonmodifiable factors. TNBC risks are greater among younger women (< 40 years) and higher among African‐American and Hispanic women. Mutations in BRCA1/BRCA2 genes are strongly associated with TNBC; > 85% of BRCA1 germline mutants display triple negative phenotype [5]. Sporadic TNBC may also occur by epigenetic inactivation of BRCA1, leading to the concept of “BRCAness,” meaning a shared phenotype with BRCA1 mutation tumors. We discuss this and other epigenetic alterations in the next section, but together they account for a large proportion of TNBCs, and are associated with aggressive clinicopathological features.

### 1.2. Molecular Differentiation of TNBC

TNBC is defined as a breast cancer where < 1% of tumor cells express ER and PR, as determined by immunohistochemistry (IHC). However, within this subset, TNBC actually covers several variants widely differing in genetic, transcriptional, histological, and clinical aspects, their common phenotype being aggressive clinical behavior. The variation is clinically relevant as it leads to differences in response to neoadjuvant therapies (NATs) and frequency of relapse [6]. They are majorly high‐grade invasive ductal carcinomas of no special type, with multiple reports colluding that they originate from ER/PR‐negative luminal stem/progenitor cells [5].

Lehmann et al. [12] classified TNBC variants into subtypes based on gene expression patterns (Table 1). It is known that BRCA1 mutation related cancers largely have a basal‐like phenotype, including absence of hormone receptors, TP53 gene mutations, and high‐level expression of proliferation‐related genes. This phenotype, found in 50%–75% of TNBCs, shows aggressive tumor behavior with limited therapy response [10]. Burstein et al. [13] differentiated breast tumors on the transcriptomic basis (Table 2). Although BLIA subtype is the most prevalent in primary tumors, LAR subtype shows the highest propensity towards lymph node metastases. The M and LAR subtypes are also reported to show a greater frequency of metastases in the bone and lungs [14].

**Table 1:** Classification of TNBC subtypes by Lehmann based on gene expression.

Lehmann classification	Key biological features	Therapeutic implication
Basal‐like 1 (BL‐1)	• High expression of cell cycle regulation and DDR genes, genetic alterations like amplifications in MYC, PIK3CA, and CDK6, and deletions in BRCA2, PTEN, MDM2, RB1, and TP53, increased expression of proliferation genes [7, 8]	• Responds to PARPi and other DNA‐damaging agents targeting defective DNA repair due to homologous recombination deficiency [7, 8]
	• Highest pCR rates for BL1 (65.6%) [9]	• Associated with the best prognosis among subtypes [9].

Basal‐like 2 (BL‐2)	• High growth factor and metabolic pathway activity with increased myo‐epithelial marker expression (TP63 and MME) as well as glycolysis, gluconeogenesis [7, 8, 10]	• May respond to immune checkpoint inhibitors, mTOR inhibitors, and growth factor inhibitors
	• Enriched in growth factor receptors like EGFR, MET, and EPHA2, TGF‐*β*, Wnt/*β*‐catenin, and IGF‐1R	• Worst prognosis [9], higher risk of recurrence

Mesenchymal (M)	• Higher EMT, cell motility, extracellular matrix remodeling, and growth factor pathway genes (PDGFR, c‐Kit, and IGF1)	• Highly aggressive, frequent lung metastasis
	• Lower proliferation rates [11]	• Potential targets include PI3K/mTOR and Src inhibitors [11]

LAR (luminal androgen receptor)	• Upregulation of hormonally regulated pathways–steroid synthesis, porphyrin metabolism, genes involved in androgen/estrogen metabolism [11]	• Relapse‐free survival comparable with BLIA subtype
	• Shows amplification of CCND1, FGF family, and MDGA2, deletion in RAD17, ERBB family, and CCNT1, aberrant expression of estrogen‐regulated genes (PGR, FOXA, XBP1, and GATA3.) and ESR1 [7]	• Lowest rate of pCR to standard chemotherapy (21.4%) subtypes [9], but best overall survival among some subtypes
		• May benefit from AR antagonists and MUC1‐targeted vaccines [7]. For some LAR subtypes checkpoint inhibitors like CDK4/6 inhibitors may be useful [7]

Abbreviations: DDR, DNA damage response; EMT, epithelial to mesenchymal transition; MME, membrane metalloendopeptidase; PARPi, PARP inhibitor; pCR, pathologic complete response; TP63, tumor protein 63.

**Table 2:** Classification of TNBC subtypes by Burstein based on transcriptome.

Burstein classification	Key biological features	Therapeutic implication
Basal‐like immune activated (BLIA)	• Upregulated immune regulation pathways (B/T/NK cell genes, STAT pathways), CDK1 upregulation [7, 11]	• STAT inhibitors
	• Generally smaller in size (pT1) with higher density of TILs, indicating immune engagement	• Antibodies to cytokines or cytokine receptors
	• BLIA tumors have the best outcome	• CTLA4 inhibitor [7, 11]
	• Lowest recurrence among BL subtypes	

Basal‐like immune suppressed (BLIS)	• Exhibits downregulation of B, T, and NK cell immune‐regulating pathways and cytokine pathways, low expression of molecules controlling antigen presentation, immune cell differentiation	• SOX inhibitors
	• Upregulated Expression of multiple SRY‐box (SOX) family transcription factors [7, 11]	• Immune‐based strategies (antibodies to PD1 or VTCN1) [7, 11]
	• Increased tumor size (pT2‐pT3)	• Combination chemotherapy, like DOX and AC, or docetaxel in combination with AC, as well as platinum‐based compounds like carboplatin and PARPi
	• BLIS tumors have the worst outcome [11]	

Mesenchymal (MES)	• Shows gene expression related to cell cycle regulation, mismatch repair, and DNA damage response, along with features of metaplastic carcinoma [7, 11]	• Inhibitors of *β*‐catenin, IGF, and PDGFR [7, 11]
	• Associated with increased aggressiveness, overexpression of genes typically associated with osteocytes and adipocytes, along with essential IGF‐1	• STAT3 inhibitors [7]
	• Pathologically, MES lacks luminal differentiation markers, linked to worse prognosis compared with other subtypes [7, 11]	

LAR (luminal androgen receptor)	• Similar to Lehmann LAR subtype, these are characterized by AR expression and hormone‐related pathways	• Therapeutical strategies for the Burstein LAR subtype align with Lehmann LAR recommendations
	• They show frequent PIK3CA mutations and low levels of TILs and Ki‐67 expression	• For AR‐negative patients and exhibit MUC1 overexpression, MUC1 vaccine advised in addition to AR antagonists [7, 11]
	• Amplification of CCND1, FGF family, MDGA2 genes, deletion in RAD17, ERBB family, and CCNT1 [7, 11]	

Abbreviations: AC, cyclophosphamide; DOX, doxorubicin; IGF‐1, insulin‐like growth factor‐1; NK, natural killer; TIL, tumor infiltrating lymphocyte.

Comparing the Lehmann and Burstein classification of basal‐like cancers, both BL‐1 and BLIA are characterized by high proliferation and basal‐like gene expression, but they differ in immune involvement [15]. BL1 tumors emphasize cell cycle regulation, DNA damage response (e.g., ATR/BRCA pathways), and proliferation markers like Ki67. BLIA tumors show upregulated immune pathways, including B/T/natural killer cell functions and STAT signaling, alongside basal‐like traits [16]. BL1 from Lehmann overlaps with both Burstein′s BLIA (immune‐activated) and BLIS (immune‐suppressed), as BL1 and BL2 split across these based on immune gene activation levels [17, 18]. BLIA often shows better prognosis than BLIS, linked to immune activation, whereas BL1 has high chemotherapy response due to proliferation vulnerability. Thus, BL1 tumors with BLIA‐like immune features may benefit from immunotherapy alongside DNA repair‐targeted therapies [7, 8].

TNBC tumors as a collective show complex genomics, with marked gene copy number alterations (CNAs) and a higher number of somatic mutations in genes like TP53 (> 80%) and PIK3CA compared with other breast cancer subtypes; Derakhshan and Reis‐Filho [5] have associated TNBC variant subclasses with CNAs in specific genes. More than 60% of TNBC cases are associated with homologous recombination repair (HRR) deficiency mutations; this has been a factor for treatment with DNA alkylating agents like platinum salts, although the results are not yet conclusive [19].

Go and Oh′s [20] review nicely summarizes the role of epigenetic alternations in TNBC—including DNA methylation, histone modifications, and noncoding RNA mediated gene silencing—and how they impact clinical management. HOX family genes, over‐expressed in TNBC, are associated with poor prognosis; this is believed to be on account of hypomethylation of promoter sequences in HOX gene clusters, which leads to an uptick in the expression of downstream genes related to metastatic phenotype. BRCA1 methylation, a “BRCAness” factor leading to its reduced expression, is another factor associated with TNBC occurrence, and it has been reported to be gender‐specific [21]. Poly‐ADP ribose polymerase (PARP) inhibitors and DNA methyltransferase inhibitors, which impact BRCA‐associated DNA damage, are being tested in combination for use in BRCA1/2 subsets of TNBC patients.

Histone modification patterns are reported to vary across TNBC subtypes. Histone deacetylase inhibitors have been shown to induce cell cycle arrest/apoptosis by promoting tumor suppressor genes or inhibiting expression of mutant growth regulatory genes. Increased activity of histone methyltransferase hSETD1A on histone segments of matrix metalloproteinase genes has been related to poorer survival characteristics in TNBC patients [22].

Noncoding microRNA sequences like miR‐638, miR‐140, miR‐489 impact chemotherapy resistance in TNBC via multiple mechanisms, including autophagy, DNA repair, transition towards metastasis, and regulation of stemness. Long noncoding RNA sequences like H19, HISLA, and NEAT1 work at the transcriptional level and are known to increase chemoresistance by inhibition of apoptosis‐related proteins, stabilization of HIF‐1*α*, and enhancement of cancer cell stemness, respectively [20].

Clinical management of TNBC thus requires knowledge of the tumor molecular profile and a patient personalized therapy approach. The main body of this review focuses on the current available modalities and emerging targeted strategies for this application.

## 2. Management of TNBC—Current and Emerging Approaches

### 2.1. Current Diagnostic and Treatment Modalities for TNBC

Breast cancer therapy shows maximum responsiveness with early detection. However, TNBCs in initial stages often exhibit benign morphologic imaging features. Thus, mammography, a common breast cancer screening technique, can give suboptimal results; even ultrasound may not correctly diagnose TNBC. Currently, breast MRI is regarded as the most sensitive detection technique, but it is not a routine screening method, more likely recommended in patients with a family history of breast cancer with or without proven BRCA mutations [23].

**Figure 1:**
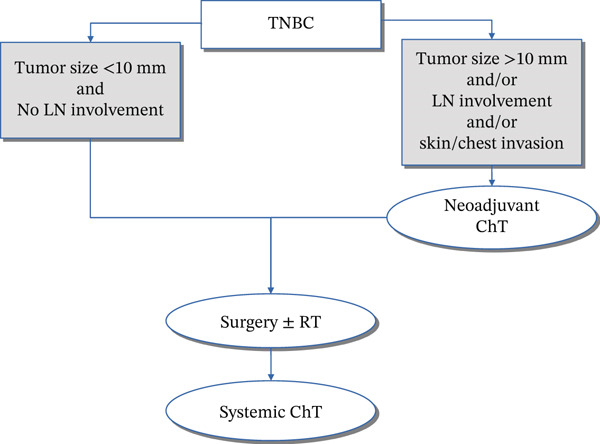
European Society for Medical Onclogy (ESMO) guidelines as applicable to triple‐negative breast cancer (LN, lymph node; ChT, chemotherapy; RT, radiotherapy).

Figure 1 illustrates European Society for Medical Oncology (ESMO) clinical practice guidelines for early breast cancer treatment as applicable to TNBC [23]. Presurgery NAT is recommended when the breast tumor is > 10 mm or an aggressive phenotype is observed or axillary involvement is observed. TNBC cases typically involve one or more of these signs and require NAT, via cytotoxic drugs or immunotherapy formulations. Such therapy can help downstage the tumor to an extent where conservative breast surgery can be recommended in place of drastic measures like mastectomy, with associated physical and psychological collateral effects.

There is significant consensus for application of NAT to treat Stage II/III TNBC [24]. The goal is to achieve complete pathologic response (cPR), meaning no residual cancer in the breast and lymph nodes after NAT. This is associated with longer overall survival (OS) and disease‐free survival (DFS). Conventional NAT includes chemotherapy with Pt‐containing compounds—taxanes and anthracyclines—and radiotherapy.

Two decades ago, chemotherapy was the only available systemic therapy for TNBC. Chemo‐resistance and toxicity of individual drugs at pharmacological concentrations were limiting factors that led to the development of combination approaches to achieve similar or better tumor killing with reduced side effects. Multiple studies have shown that incorporation of carboplatin to a paclitaxel and anthracycline‐based treatment improved cPR in TNBC patients. However, at least one large study (720 patients) suggests that women > 50 years do not benefit from this combination in terms of cPR, OS, or DFS [25]. Taking into account the serious side effects of carboplatin [24], necessary caution may be applied while recommending it as standard care. A 2022 meta‐analysis of randomized controlled trials of capecitabine—a prodrug of fluorouracil—showed improved DFS and OS with tolerable side effects as adjuvant therapy for TNBC [26].

Radiation therapy causes cytotoxicity by DNA damage, mainly as double‐strand breaks (DSBs). Complete breast radiotherapy as an adjunct to early breast‐conserving surgery has brought a fivefold reduction in local recurrence of breast cancer overall. In a study with early‐stage TNBC patients, the rate of DFS at more than 7 years for patients with conservative breast surgery and radiotherapy against radical mastectomy without radiotherapy was 94% and 85%, respectively [27]. Recommendation for radiotherapy postradical mastectomy is based on lymph node staging. TNBC resistance to radiotherapy in comparison with luminal breast cancers (which express hormone receptors) has been linked to biomarkers like epidermal growth factor receptor (EGFR), maternal embryonic leucine zipper kinase, and PARP. These can be considered factors to modify sensitivity to radiotherapy. Interestingly, a study on the association between androgen receptor (AR) expression and radioresistance in TNBC has implied that ionizing radiation induces AR expression in TNBC. AR expression is positively correlated with radioresistance, possibly by more effective DSB repair. Targeted inhibition of AR function may improve response/reduce resistance to radiation therapy [28]. In contrast, Spurnić et al.′s [29] study of 124 early‐stage TNBC patients concludes that AR positivity is an independent favorable marker for 5‐year survival following surgical treatment. The jury is therefore still out on AR expression and its relevance to TNBC prognosis. In general, a greater understanding of molecular targets in TNBC has led to the development of new therapies that may be used instead of or in combination with conventional NATs to achieve better response with reduced collateral damage. Molecular markers capable of differential diagnosis of TNBCs can also be used to make treatment decisions. Burstein′s [13] subtyping of TNBCs allows for selection of appropriate NATs for the various groups, which show differential response to chemotherapy, local and distant disease progression, and prognosis. As per this classification, the BLIS cluster showed the least favorable scenario in terms of DFS, indicating a significant role for immune response in TNBC treatment. BRCA1 mutant TNBC patients have a favorable overall response rate even with a single dose of cisplatin, and patients with basal‐like tumors but high immune response displayed improved DFS over those with low immune response [30]. Knowledge of the TNBC subtype can thus benefit treatment decision‐making. There are other TNBC molecular markers in various stages of clinical and preclinical study. We will explore some of these in detail in the next section.

### 2.2. Emerging Molecular Markers for TNBC Diagnosis and Therapy

**Figure 2:**
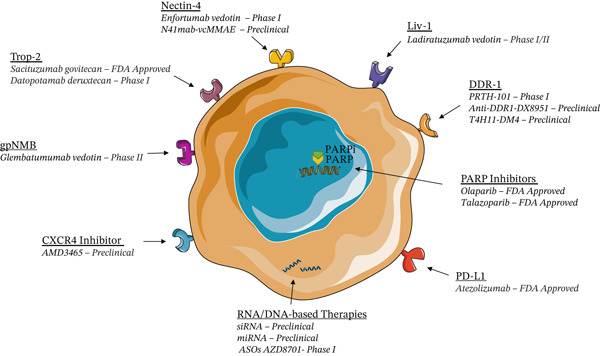
TNBC‐associated markers and corresponding targeted therapeutics (Image created from source files hosted by Servier Medical Art [https://smart.servier.com], licensed under CC BY 4.0 [https://creativecommons.org/licenses/by/4.0/]).

Figure 2 gives a representation of various emerging biomarkers for TNBC, and lists some of the corresponding candidates for its targeted therapy. These will be discussed in detail in this section.

#### 2.2.1. Trophoblast Cell‐Surface Antigen (Trop2)

Trop2 is a 323 amino acid transmembrane glycoprotein associated with cell migration and anchorage‐independent growth. In breast cancer, it impacts tumor growth, invasion, metastasis, and treatment resistance. Trop2 expression is increased by up to 93% in TNBC as assessed by IHC staining [31]. Several anti‐trop2 monoclonal antibodies and antibody–drug conjugates (ADCs) are in clinical use. We now discuss their current status in TNBC therapy.

Sacituzumab govitecan (SG) (Trodelvy/IMMU‐132) is an FDA‐approved ADC for treatment of trop2 positive metastatic TNBC. Antineoplastic drug SN‐38 attached to the antibody effects cytotoxicity of the ADC: An active metabolite of topoisomerase I inhibitor irinotecan, SN‐38 prevents DNA replication in cancer cells by preventing rejoining of cleaved strands, thus causing cell death [32]. Multiple clinical trials have shown that SG is promising for TNBC therapy: NCT01631552 (IMMU‐132‐01) has shown 33.3% overall response rate (defined as the proportion of patients with partial/complete response to therapy), mean progression‐free survival (mPFS) of 5.5 months and OS increased to 13.0 months in patients with metastatic TNBC. NCT02574455 evaluated the potential of SG versus treatment of physician′s choice (i.e., eribulin, capecitabine, gemcitabine, or vinorelbine) in TNBC, and found that with SG, mPFS was 5.6 months and median overall survival (mOS) of 12.1 months, whereas with TPC the mPFS was 1.7 months and mOS only 6.7 months [33]. Datopotamab deruxtecan (DS‐1062) is another ADC targeting trop2; it is under investigation for treating metastatic breast cancer. In June 2025, this ADC received FDA approval for adults with locally advanced or metastatic EGFR‐mutated nonsmall cell lung cancer [34]. Phase I clinical trial TROPION‐PanTumor01 (NCT03401385) to study DS‐1062 in advanced solid tumors including TNBC is expected to be complete by 2026.

#### 2.2.2. Nectin‐4

Nectin‐4 is a Ca^+2^‐independent immunoglobulin‐like protein coded by a poliovirus receptor‐like 4 (PVRL‐4) gene. There are four nectins in humans, Nectin‐1 to Nectin‐4. Nectin‐4 is expressed in embryonic and placental tissues with no/low expression in adult tissue. However, its expression is increased in several cancers, including breast [35]. Nectin‐4 is associated with the development of tumor characteristics like adhesion, migration, and angiogenesis. Nectin‐4 targeting enfortumab vedotin (EV) is an FDA‐approved ADC for urothelial carcinoma. A Phase‐II clinical trial EV‐202 (NCT04225117) is ongoing to evaluate its potential in treating locally advanced or metastatic solid tumors including TNBC [36]. A detailed discussion on Nectin‐4 and its role in targeting different cancers is provided in a recent review by Li et al. [37]. Here, we discuss Nectin‐4 in TNBC.

Nectin‐4 is reportedly overexpressed in 62% TNBC cases, its higher expression associated with poor prognosis. In a 2016 study, the ADC N41mAb‐vcMMAE—comprising of human anti‐nectin‐4 monoclonal antibody (mAb) conjugated to monomethyl auristatin‐E (MMAE)—was found to effectively target Nectin‐4 TNBC cell lines in vitro and effect a complete and durable response in Nectin‐4 positive TNBC xenografts. The study concluded that Nectin‐4 can be a prognostic biomarker and specific therapeutic target for TNBC [38]. However, a conflicting finding associated high Nectin‐4 expression with a better OS in TNBC. This study used protein expression by IHC as compared with mRNA expression analysis by the previous group [39]. As IHC measures actual receptor concentration, it may represent a more reliable clinical hypothesis.

Shao et al. developed a theranostic pair consisting of a mAb–based radio‐imaging agent, ^99m^Tc‐HYNIC‐mAb_Nectin-4_ for TNBC diagnosis and mAb_Nectin-4_‐indocyanine green (ICG) to mediate photo‐thermal therapy of TNBC [40]. Duan et al. recently developed a bicyclic peptide‐based radiotracer, ^68^Ga‐N188, for positron emission tomography (PET) imaging of membranous Nectin‐4 expression that can help in patient stratification and treatment selection for EV. They compared the results of ^68^Ga‐N188 with ^18^F‐FDG PET scan and found that in multiple cancers including TNBC, the detection rate of the tracers was comparable (95.00% vs. 93.33%) [41]. Babekar et al. have reported another theranostic pair using ^225^Ac and ^89^Zr‐labeled anti‐nectin‐4 N4MU01 radio immunoconjugates in preclinical TNBC xenograft and syngeneic mice models. [^89^Zr]Zr‐DFO‐N4MU01 in mice bearing triple‐negative MDA‐MB‐468 xenografts showed tumor uptake of 13.2*%* ± 1.12*%* injected activity per gram at 120 h postadministration, whereas mice bearing MDA‐MB‐468 xenografts and 4 T1_.nectin-4_ syngenic grafts treated with alpha‐emitting [^225^Ac]Ac‐Macropa‐N4MU01 showed 100% (6/6) and 83.3% (5/6) tumor remission, respectively [42].

#### 2.2.3. LIV‐1

LIV‐1 is a multispan transmembrane protein with metalloproteinase and zinc transporter activity, overexpressed in malignancies, including metastatic ER positive and TNBC. High LIV‐1 expression is linked to cancer cell motility, histological grade, and early node metastasis. LIV‐1 is expressed in 65% of TNBC patients [32].

Ladiratuzumab vedotin (SGN‐LIV1A) is an ADC composed of (a) humanized antibody targeting LIV‐1, (b) cytotoxic drug MMAE, and (c) proteolytically cleavable linker. It is under multiple Phase I/II clinical trials (NCT03424005, NCT03310957, and NCT01042379) for treatment of TNBC and other solid tumors [32].

#### 2.2.4. Glycoprotein Nonmetastatic B (gpNMB)

gpNMB/osteoactivin is a transmembrane protein overexpressed in various cancers—including 30%–40% of TNBC cases—and is linked to an unfavorable prognosis [43]. In various preclinical studies, the marker has been associated with tumor cell invasion, metastasis, and angiogenesis [44]. Humanized glembatumumab binds to gpNMB expressing cells and internalizes into their lysosomal compartment. When coupled with a cytotoxic drug like the microtubule inhibitor MMAE, it forms an ADC (glembatumumab vedotin) that can affect targeted therapy. The Phase II study NCT01997333, however, showed no significant advantage in progression‐free survival (PFS) over the chemotherapeutic prodrug capecitabine [44], and further clinical development was discontinued. In animal studies with MDA‐MB‐468 xenografts, the kinase inhibitor dasatinib has been observed to upregulate gpNMB expression with the aim of increasing gpNMB‐targeted tumor cell death [45].

#### 2.2.5. Chemokine Receptor Type 4 (CXCR4)

CXCR4 is connected to the inside of the cell membrane via intracellular G‐protein. CXCR4 activation triggers a cascade of events associated with tumor progression, including transcription control, Ca^++^ mobilization triggering, and rearrangement of actin filaments to enhance invasion and chemotaxis. CXCR4 overexpressing cells can initiate tumors in mouse models of various cancers. They are regarded as cancer stem cells; their actions not only induce proliferation but also resistance to chemo/radiotherapy induced cell death [46]. Peptides derived from horseshoe crab origin polyphemusin II have been tested for anti‐CXCR4 activity. Preclinical studies with CTCE‐9908, a peptide analog of CXCR4 binder CXCL12, showed multifold reduction in breast tumor size. Monomacrocylic high affinity CXCR4 inhibitor AMD3465 inhibited breast tumor growth and lung/liver metastases in preclinical tumor models [46]. Bao et al. [47] report a synergistic combination of CXCR4 antagonist with fibroblast activation protein (FAP) therapeutic radiopharmaceutical [^177^Lu]Lu‐DOTAGA.(SA.FAPi)_2_. Among small molecules, the immunosuppressant drug everolimus has been observed in preclinical studies to mitigate invasive and metastatic behavior of TNBC cells by decreasing CXCR4 expression [48].

#### 2.2.6. Discoidin Domain Receptor 1 (DDR1)

DDR1 is a transmembrane receptor tyrosine kinase, overexpressed in multiple solid tumors including TNBC, while rarely expressed in normal tissue. Tao et al.′s [49] study of 140 breast cancer cases found high DDR1 levels in > 60% of tissue samples; additionally, the frequency of overexpression correlated with stage advancement. DDR1 is linked to metastatic behavior and poor disease prognosis. It has been reported to protect tumor cells from infiltration by immune cells by promoting collagen fiber alignment [50]. DDR1 overexpression is also correlated with Src and FAK activation, which have known activity in cancer progression [51]. Thus, it is an important breast cancer biomarker. NCT05753722, a Phase 1 clinical study of anti‐DDR1 humanized antibody PRTH‐101 in metastatic solid tumors, is currently underway; in preclinical animal tumor models, this antibody was shown to disrupt collagen fiber alignment, thus facilitating tumor infiltration by CD8+ T‐cells [52]. The ADC anti‐DDR1‐DX8951 is currently in preclinical trials and has demonstrated antitumor activity in TNBC xenograft mouse models [53]. T_4_H_11_‐DM4, an ADC combining DDR1 targeting murine mAb with an antitubulin drug, has shown receptor specific cytotoxicity in vitro and antiproliferative activity in DDR1‐expressing breast tumor xenograft mouse models [49]. Whether it can be successfully humanized and give similar results in actual patients remains to be seen. Interestingly, other studies have associated DDR1 with reduced cell proliferation and apoptosis induction in breast cancer [54].

#### 2.2.7. Programmed Death Ligand 1 (PD‐L1)

PD‐L1 is an immune checkpoint protein expressed by antigen presenting cells to prevent over‐activation of T‐cells. When expressed on cancerous cells or immune cells, it interacts with PD‐1 receptor on T‐lymphocyte surface, leading to evasion of T‐cell cytotoxicity. Further, it has been observed that modification of tumor microenvironment (TME) by increased deposition of extracellular matrix (ECM) activates pathways causing elevated expression of PD‐L1, indicating that it is a precursor to metastasis [55]. In TNBC patients, the tumor‐infiltrating leukocytes (TILs) have higher PD‐L1 expression than tumor cells, thus inhibiting anticancer immune response [56]. Therefore, inhibition of either PD‐1 or PD‐L1 has emerged as a promising strategy in multiple solid tumors. PD‐L1 is expressed in 20‐30% of TNBCs [57], and blocking these ligands or the PD‐1 receptor reactivates the cytotoxic T‐cells. FDA approved anti–PD‐L1 atezolizumab in 2016 and anti–PD‐1 pembrolizumab in 2018 for cancer immunotherapy. Recently, the combination of atezolizumab with nanoparticle albumin‐bound (nab)‐paclitaxel has received FDA approval for treating PD‐L1 positive metastatic TNBC. According to the second interim OS analysis of the IMpassion130 Phase III trial, mOS was 21.0 months in the atezolizumab plus nab‐paclitaxel group versus 18.7 months in the placebo plus nab‐paclitaxel group. This difference in OS is not significant between the two groups. However, in patients with PD‐L1 immune cell‐positive tumors, mOS was 25·0 months with atezolizumab plus nab‐paclitaxel group versus 18·0 months with placebo plus nab‐paclitaxel group [58]. An evaluation of Phase III trials comparing pembrolizumab plus chemotherapy group (nab‐paclitaxel, paclitaxel, or gemcitabine–carboplatin) and placebo‐chemotherapy group in “CPS 10” patients—that is whose combined positive score (CPS) for PD‐L1 expression ≥ 10—concluded that mOS was 23.0 months in the pembrolizumab–chemotherapy group and 16.1 months the placebo–chemotherapy group [59]. This is a difference of > 40%, showing clear benefit of pembrolizumab therapy and justifying the accelerated approval for its clinical use in 2020 by the FDA [60].

#### 2.2.8. Poly‐ADP Ribose Polymerase 1 (PARP1)

PARP1 is an intracellular enzyme involved in base excision repair pathway of single stranded DNA breaks. Poly‐ADP ribose polymerase inhibitors (PARPi) block this function, causing DNA damage accumulation and eventual cell death [61]. Presently, there are two FDA‐approved PARPi for metastatic TNBC—olaparib and talazoparib (TAL) [62]. They have shown effectiveness in killing cancer cells with BRCA 1/2 mutations by synthetic lethality, a type of genetic interaction whereby simultaneous inactivation of both genes leads to cell death. Owing to their heterogeneous nature TNBCs develop resistance to PARPi by different mechanisms, including HRR restoration by gene reversion, stabilization of replication forks from reduced recruitment of nucleases, reduced PARP1 trapping and drug efflux [63]. Several clinical trials are exploring strategies to overcome PARPi resistance through combination therapies with ataxia telangiectasia and Rad3‐related protein (ATR) inhibitors (VX‐970 [berzosertib], AZD6738 [ceralasertib], VX‐803 [gartisertib], BAY1895344 [elimusertib {ELI}], and RP‐3500 [camonsertib]) or CHK1 inhibitors (e.g., SRA737 and prexasertib). ATR, a key regulator of genome integrity contributes to PARPi resistance particularly in homologous recombination deficient tumors. ELI when combined with TAL partially reversed TAL resistance in TNBC models: In vitro studies with TAL‐resistant cell lines showed < 12.5% inhibition of cell viability even at 10‐nM TAL concentration for up to 12 days, whereas the combination of 1 nM ELI + 8 nM TAL reduced cell viability to 50.3% within 72 h of incubation [64, 65]. CHK1 inhibition amplifies DNA damage by blocking the S/G2 checkpoints induced by PARPi, suppressing RAD51, and increasing DNA damage markers such as *γ*H2AX and phosphorylated replication protein A (RPA). A 2024 study showed regression of PARPi‐resistant ovarian cancer xenografts using a combination of CHK1 inhibitor SRA737 and PARPi, with partial response observed in BRCA1/2 mutant resistant tumors during Phase I trials, but for TNBC it is yet to be tested [66]. The combination of prexasertib and olaparib in a Phase II clinical trial demonstrated only 11% objective response rate in patients with BRCA wild‐type TNBC [67].

#### 2.2.9. EGFR

In breast cancer, EGFR signaling contributes to tumor progression, invasion, metastasis, and therapeutic resistance [68]. Reported rates of EGFR overexpression in TNBC vary substantially—from 40% to 89% depending on the source—due to differences in scoring systems, antibody selection, and patient demographics [69, 70]. No EGFR‐targeted therapies have yet been approved for TNBC. However, multiple anti‐EGFR monoclonal antibodies and ADCs are under clinical/preclinical investigation. A Phase II trial (NCT02593175) where anti‐EGFR panitumumab combined with carboplatin and paclitaxel was evaluated as a second phase NAT in patients with doxorubicin and cyclophosphamide‐resistant TNBC showed a pathological complete response (pCR) rate of 30.4% (13/43 patients) [71], indicating modest effectiveness in a resistant setting. Another Phase II study of doxorubicin‐loaded anti‐EGFR immunoliposomes (NCT02833766) in advanced EGFR‐positive TNBC failed to meet its primary endpoint of 12‐month PFS despite an acceptable safety profile, leading to discontinuation of further development [72]. In preclinical studies, Cheung et al.′s [73] anti‐EGFR cetuximab conjugate with CDK inhibitor SNS‐032 demonstrated growth suppression in spheroid and xenograft TNBC models. Recently, EGFR blocker regorafenib inhibited tumor metastases in EGFR‐expressing TNBC models during preclinical studies [74].

#### 2.2.10. RNA/DNA‐Based Therapies

RNA‐based therapies and gene therapies are emerging as potential TNBC treatments [75]. RNA‐based therapies including small interfering RNA (siRNA), microRNA (miRNA), antisense oligonucleotides (ASO) use RNA molecules to modulate gene expression and cellular function [76]. siRNAs are short double‐stranded RNA molecules that can selectively silence gene expression by targeting the corresponding messenger RNA (mRNA). Subhan and Torchilin′s [77] comprehensive review of siRNA usage in TNBC therapy lists several examples of genes involved in breast cancer pathways—like ATP‐binding cassette B1 involved in multidrug resistance, and the apoptosis inhibitor survivin being downregulated using siRNA. miRNAs are small noncoding RNAs that regulate gene expression by binding to mRNA and inhibiting translation or promoting degradation. Multiple preclinical miRNA candidates are targeting TNBC genes related to proliferation, metastasis, and invasion [76]. These include miR‐143, which targets the Muc1 gene leading to suppression of cell proliferation and miR‐708 coated on gold nanoparticles, observed to reduce lung metastasis of TNBC. ASOs are short, synthetic, single‐stranded DNA/RNA sequences designed to bind with specific RNA molecules, thereby altering their function. Currently in Phase I trial (NCT04504669), AZD8701 from AstraZeneca reduces mRNA expression of FOXP3 protein. This protein governs development and function of regulatory T‐cells known to dampen immune response against cancer cells. Thus, the ASO ensures activated anti‐tumor immune response. [78].

CpG oligonucleotides are synthetic DNA molecules that mimic bacterial DNA and stimulate immune response. They have been shown to remodel macrophage metabolic pathways and enhance their anti‐tumor activity. However, due to their negative charge, CpG oligonucleotides are not efficiently endocytosed by immune cells. To overcome this limitation and improve both loading capacity and selective targeting of immune cells, Gao et al. [75] developed a novel Mn^+2^‐functionalized covalent organic framework (MnCOF) to carry CpG oligonucleotides inside tumor‐associated macrophages (TAMs) for treatment of metastatic TNBC. These MnCOF‐CpG engineered macrophages not only suppressed metastasis; they also relieved tumor hypoxia, prevented immune cell exhaustion, and enhanced CD8^+^ T‐cell infiltration, resulting in effective inhibition of TNBC growth and distant metastasis [79].

### 2.3. Molecular Modulation of TME

Fertal et al.′s [55] review says that the TME or stroma has a profound impact on the perceived heterogeneity of TNBC, and the study of cancer tissue interactions with the TME is an important aspect of developing effective treatment strategies. Increased deposits of ECM in breast tumors and alterations in tumor‐associated collagen signatures are said to indicate a poor prognosis as they result in stiffness that leads to poor diffusion, a greater degree of hypoxia and metabolic stress. This in turn causes reduced infiltration of immune cells leading to immunosuppression as well as resistance to immune checkpoint inhibitor (ICI) therapies, and also inhibits the penetration of chemotherapy agents, thereby reducing their efficacy. Fibrotic ECM also activates signaling in cancer‐associated fibroblasts, a precursor to infiltration by protumor immune cells. TNBC targeting ADCs like SG show limited diffusion in the presence of fibrotic ECM; the use of collagen inhibitors was shown to increase ADC penetration in preclinical experiments, though it has not successfully translated to the clinic. Variations in perfusion to the tumor tissue can also cause differences in effectiveness of treatment of solid tumors, including TNBC. The activity of ECM modulator losartan in conjunction with PD‐1 inhibitor camrelizumab and liposomal doxorubicin is currently in Phase II clinical trials (NCT05097248). Apart from suppression of antitumor defenses, the stiffening of the TME is associated with the active diversion of prometastatic TAMs and regulatory T‐cells to the primary lesion, thereby actively promoting metastasis. Rebastinib, a targeted inhibitor of Tie2 receptor expressing TAMs, in conjunction with chemotherapy is in Phase I trials for metastatic breast cancer patients.

Table 3 lists several of the ongoing clinical trials for evaluating newer therapeutic approaches to TNBC.

**Table 3:** Overview of ongoing clinical trials for TNBC management.

Start—end date phase	Recruitment status total patients	Drug regimen	Trial objective	Trial number [reference]
2021–27 Phase 1/2, open label.	Recruiting 63	SINE selinexor + PARPi talazoparib	Determine safety profile and recommended Phase 2 dose of drug combination for LA/mTNBC	NCT05035745 [80]
2024–28 Phase 2, multicenter.	Recruiting 53	CDK4/6i abemaciclib + AR‐blocker bicalutamide	Determine safety and utility of combination for AR‐positive mTNBC	NCT06365788 [81]
2024–27 Phase 2, open label.	Active, not recruiting 36	CDK4/6i trilaciclib + ICI PEMBRO + nucleoside analog GEM + alkylating agent CBDCA	Evaluate efficacy in LA/mTNBC	NCT06027268 [82]
2024–27 Phase 3, randomized, open label, multicenter.	Active, not recruiting 350	Anti‐trop2 mAb FDA018‐ADC	Assess efficacy and safety over ICC in inoperable TNBC resistant or recurring after taxane therapy	NCT06519370 [83]
2022–31 Phase 3, open label, randomized.	Recruiting 1514	Anti‐trop2 ADC IMMU‐132 + ICI PEMBRO versus TPC (PEMBRO +/− CAP)	Assess safety and efficacy of IMMU‐32 + PEMBRO against TPC for residual TNBC post‐NAT/surgery	NCT05633654 [84]
2022–28 Phase 3, open label, randomized.	Active, not recruiting 623	Anti‐trop2 ADC IMMU‐132 versus TPC (PTX, nab‐PTX, GEM, and CBDCA)	Compare PFS between IMMU‐32 and TPC for PD‐L1 negative LA/mTNBC	NCT05382299 [85]
2025–30 Phase 3, randomized, open label.	Recruiting 1000	Anti‐trop2 ADC Sac‐TMT +/− ICI PEMBRO versus TPC (Rescue medication, PTX, Nab‐PTX, GEM, and CBDCA)	Comparison of survival of PD‐L1 negative LR/mTNBC patients treated with targeted ligands versus standard chemotherapy	NCT06841354 [86]
2025–29 Phase 2	Not yet recruiting 41	Anti‐trop2 ADC Sac‐TMT + toripalimab	Evaluate safety and efficacy for first line treatment of PD‐L1 positive LA/mTNBC	NCT07244874 [87]
2018–27 Phase 1, multicenter, open label.	Active, not recruiting 890	Anti‐trop2 ADC DS‐1062a	Assess dose escalation and expansion in patients with NSCLC and TNBC resistant/ineligible for standard treatment	NCT03401385 [88]
2023–26 Phase 3	Recruiting 192	VEGFRi BP102 + nab‐PTX/TPC versus nab‐PTX versus TPC	Evaluate addition of VEGFRi to nab‐PTX/TPC in first‐line treatment of TNBC BLIS subtype	NCT05806060 [89]
2024–27 Phase 1a/1b multicenter, open‐label.	Recruiting 420	Anti‐nectin‐4 ADC LY4052031	Determine safety and efficacy in patients with nectin‐4 expressing LA/metastatic solid tumors (including TNBC)	NCT06465069 [90]
2020–26 Phase 2, open label, multicenter, multicohort.	Active, not recruiting 329	Anti‐nectin‐4 ADC EV +/− ICI PEMBRO	Determine Objective Response Rate to the ADC and compare safety/MTD with EV +/− PEMBRO for LA/metastatic solid tumors (including TNBC)	NCT04225117 [36]
2022–27 Phase 1b, open label, one‐center.	Recruiting 65	AR‐blocker seviteronel + synthetic glucocoticoid dexamethasone+ taxane docetaxel	Determine safety and efficacy of combination regimen in mTNBC	NCT04947189 [91]
2021–27 Phase 2	Recruiting 34	ICI PEMBRO +/‐ PARPi olaparib + radiotherapy	Assess safety and possible benefit of targeted ligands to standard radiotherapy for mTNBC	NCT04683679 [92]
2022–26 Phase 1b/2	Active, not recruiting 42	Anti‐PD‐L1/VEGF bsmAb PM8002 + Nab‐PTX	Assess safety and overall response rate of drug combination as first‐line therapy in LA/mTNBC	NCT05918133 [93]
2024–28 Phase 3, multicenter, randomized, double‐blind.	Recruiting 360	Anti‐PD‐L1/VEGF bsmAb PM8002 or placebo + Nab‐PTX	Evaluate safety and efficacy of PM8002 + Nab‐PTX as first‐line treatment for LA/mTNBC	NCT06419621 [94]
2023–26 Phase 3, open label, randomized.	Recruiting 203	MTORi everolimus + TPC (nab‐PTX, CAP, eribulin, CBDCA, vinorelbine, or utidelone) versus TPC alone	Compare efficacy of TPC alone and TPC + everolimus in treatment of LAR subtype LR/mTNBC	NCT05954442 [95]
2021–28 Phase 1a/1b, multicenter, open label.	Recruiting 345	IR modulator NX‐1607 +/− PTX	Assess safety and anticancer activity of NX‐1607 alone and in combination with PTX in advanced malignancies, including in TNBC	NCT05107674 [96]
2020–27 Phase 2	Recruiting 40	PD‐L1 blocker atezolizumab + Anti‐trop2 ADC IMMU‐132	Assess feasibility of a combination of targeted ligands for treating residual TNBC	NCT04434040 [97]
2023–27 Phase 1, open label.	Recruiting 270	Anti‐DDR1 PRTH‐101 +/− ICI PEMBRO	Evaluate safety and MTD of PRTH alone/combined with PEMBRO in LA/metastatic solid tumors (including TNBC)	NCT05753722 [98]
2023–2027 Phase 1/1b, open‐label, multicenter.	Recruiting 130	EGFR and CD3 targeting tumor‐activated IR modulator JANX008	Dose escalation and expansion study to assess safety and preliminary anti‐tumor activity in EGFR‐expressing LA/metastatic carcinoma, including TNBC	NCT05783622 [99]
2025–28 Phase 1b/2, open label.	Recruiting 50	Selective T‐cell receptor targeting bifunctional antibody‐fusion molecule STAR0602 + anti‐trop2 ADC IMMU‐132	Investigate safety and efficacy of drug combination for LA/metastatic solid tumors (including TNBC)	NCT06827613 [100]
2024–28 Phase 2, nonrandomized, multicenter.	Recruiting 60	Anti PD‐1/CTLA4 IR modulating bsmAb QL1706 + Nab‐PTX +/− anti‐VEGF BVZ	Assess safety and efficacy of drug combination as first line treatment for LR/mTNBC	NCT06786026 [101]
2021–24 Phase 2	Not yet recruiting 52	Anti‐PD‐1 camrelizumab + liposomal DOX + ARB losartan	Assess safety and efficacy of drug combination as second‐line therapy for advanced/LA TNBC	NCT05097248 [102]
2024–28 Phase 2, randomized.	Recruiting 106	PD‐L1 blocker atezolizumab+ Nab‐PTX alone versus after two cycles of PTX + BVZ	Assess efficacy of targeted drug combination in PD‐L1 positive mTNBC patients	NCT06793553 [103]
2022–27 Phase 2, open label, multiarm parallel study.	Recruiting 80	Immune microenvironment modulator cocktail (sodium cromoglicate, choline, efavirenz, SHR 1811, SHR 2102, and mecapegfilgrastim) + ICI AK131	Evaluate safety and efficacy of combination therapy in mTNBC patients that progressed during/after previous ICIs	NCT05076682 [104]
2018–24 Phase 1b/2, open label.	Completed 185	Anti‐LIV1 ADC SGN‐LIV1A + ICI PEMBRO	Assess dose escalation and objective response rate as first‐line combination therapy in LA/mTNBC	NCT03310957 [105]
2020–24 Phase 1, first‐in‐human.	Completed (not yet publicly available) 60	Immunomodulatory antisense oligonucleotide AZD8701 +/− anti‐PD‐L1 Durvaluamb	Assess safety, pharmacokinetics, pharmacodyamics and anticancer activity in select advanced solid tumors (including TNBC)	NCT04504669 [106]
2018–30 Phase 1b/2, open‐label, multicenter, randomized.	Recruiting 792	Multiple treatment combinations of chemotherapeutic agents + targeted ligands including PD‐L1 blockers, anti‐VEGF Mabs, and IMMU‐132	Assess efficacy and safety of combinations in patients with LA/metastatic breast cancer (including TNBC)	NCT03424005 [107]

Abbreviations: ADC, antibody–drug complex; ARB, angiotensin II receptor blocker; BVZ, bevacizumab; bsmAb, bispecific monoclonal antibody; CAP, capecitabine; EV, enfortumab vedotin; ICI, immune checkpoint inhibitor; IR, immune response; LA, locally advanced; LR, locally recurrent; MAb, monoclonal antibody; MED, minimum essential dose; MTD, maximum tolerated dose; mTNBC, metastatic TNBC; mTORi, mTOR inhibitor; Nab, nanoparticle albumin‐bound; NAT, neoadjuvant therapy; NSCLC, nonsmall‐cell lung cancer; PARPi, PARP inhibitor; PD‐1R, PD‐1 receptor; PEMBRO, pembrolizumab; PFS, progression‐free survival; PTX, paclitaxel; SINE, selective inhibitor of nuclear export; TNBC, triple‐negative breast cancer; TPC, treatment of physician′s choice; VEGFRi, VEGFR inhibitor.

## 3. Conclusion and Future Perspectives

As the preceding discussion in the article suggests, the initiation and spread of TNBC is a phenomenon governed by multiple factors that, in turn, impacts several other pathways and processes in the surrounding microenvironment. Stratification of TNBC patients based on known histological/molecular subtypes and expression of specific markers as previously discussed allows for individualized therapeutic approaches and increases the likelihood of improved clinical outcomes in terms of both survival (OS/DFS) and quality of life parameters. Knowledge gained in the diagnostic workflow about presence of specific markers like trop2, nectin‐4, and LIV‐1 can be used to tailor the use of specific inhibitors and ADCs that are best likely to be effective against the individual tumors. In specific terms, this could improve the effectiveness of NAT given prior to surgery by targeted blocking of prometastatic processes, allowing for easier transition to a state where surgery becomes feasible and downplaying the requirement for subsequent systemic chemotherapy. Currently, such classification is not as widely adopted in clinical recommendations. Incorporating them in a systematic manner could greatly enhance the process of therapy decision‐making and ensure that patients receive the most effective treatment. The section on emerging molecular markers points to several ongoing clinical trials testing the safety and efficacy of targeted ligands in relevant patient groups. When completed, the results of these trials can potentially show improved effectiveness of treatments that are customized towards the specific biomarker profiles of the patients. Additionally, individual therapeutic modalities may benefit only a subset of TNBC patients owing to their heterogeneity, as the efficacy depends on individual marker profiles [108]. Several clinical trials are currently underway to evaluate combinations of drugs targeted towards different molecular pathways or synergy of drugs with other modalities like radiotherapy or immunotherapy as a means of reducing the development of resistance to a single target. Another as yet largely unexplored path for TNBC treatment is the use of targeted radioligand therapy (RLT), with only one clinical report of a single TNBC patient treated with Lutetium‐177 (^177^Lu) labeled PSMA targeting ligand [109], and some preclinical animal model studies with ^177^Lu labeled gold nanoparticles, PSMA‐617 ligand, alkyl phosphocholine (NM‐600), and so on compiled in Gulec et al.′s review [110]. If stably combined with some of the TNBC specific ligands previously described, for example, anti‐trop2 sacituzumab, anti‐nectin‐4 N4MU01, anti‐PD‐L1 atezolizumab, known targeted therapy radioisotopes like ^177^Lu, ^225^Ac, and ^211^As have solid potential in clinical TNBC management. Thus, the continued process of identification and incorporation of multiple molecular targets can be expected to translate to a better arsenal of treatment options for a challenging ailment.

**Nomenclature**
ADC: Antibody‐drug conjugate
AR: Androgen receptor
BLIA/BLIS: Basal‐like immune activated/suppressed
CNA: Copy number alteration
cPR: Complete pathologic response
CPS: Combined positive score
DFS: Disease‐free survival
DSB: Double‐strand break
ECM: Extracellular matrix
ER: Estrogen receptor
ESMO: European Society for Medical Oncology
HRR: Homologous recombination repair
IHC: Immunohistochemistry
mAb: Monoclonal antibody
MnCOF: Mn^+2^‐functionalized covalent organic framework
NATs: Neoadjuvant therapies
OS: Overall survival
PARP: Poly‐ADP ribose polymerase
PFS: Progression‐free survival
PR: Progesterone receptor
TIL: Tumor‐infiltrating lymphocyte
TME: Tumor microenvironment
TNBC: Triple‐negative breast cancer

## Author Contributions

Anuj Kumar has substantially contributed to the drafting and revising of the manuscript and has prepared the figures and tables used in it. Suresh Subramanian is credited with the original concept of the review, substantial contribution to drafting and revisions, and formatting the final version for submission.

## Funding

No funding was received for this manuscript.

## Ethics Statement

The authors have nothing to report.

## Consent

The authors have nothing to report.

## Conflicts of Interest

The authors declare no conflicts of interest.

## Data Availability

Data sharing is not applicable to this article as no datasets were generated or analyzed during the current study.
